# Do weight perceptions among obese adults in Great Britain match clinical definitions? Analysis of cross-sectional surveys from 2007 and 2012

**DOI:** 10.1136/bmjopen-2014-005561

**Published:** 2014-11-05

**Authors:** Fiona Johnson, Rebecca J Beeken, Helen Croker, Jane Wardle

**Affiliations:** Department of Epidemiology and Public Health, Health Behaviour Research Centre, University College London, London, UK

**Keywords:** PUBLIC HEALTH, EPIDEMIOLOGY

## Abstract

**Objectives:**

To assess the proportion of the adult obese population in Great Britain who would describe their weight using the terms ‘obese’ and ‘very overweight’ in 2007 and 2012, and identify factors associated with more accurate weight perceptions.

**Design:**

Analysis of weight perception data from two population-based surveys.

**Setting:**

Population surveys conducted in Great Britain.

**Participants:**

Survey respondents (N=657) whose self-reported weight and height placed them in the obese category: body mass index (BMI) ≥30.

**Primary outcome measure:**

Self-identification using the terms ‘obese’ and ‘very overweight’.

**Results:**

The proportion of obese adults selecting the term ‘obese’ to describe their body size was very low in both women (13% in 2007 and 11% in 2012) and men (4% in 2007 and 7% in 2012) and did not change significantly. Recognition of a substantial degree of overweight (as indexed by endorsement of either of the terms ‘obese’ or ‘very overweight’) declined substantially in women, from 50% in 2007 to 34% in 2012. It was not significantly changed in men (27% in 2007 and 23% in 2012). Having a higher BMI, and being able to identify the BMI threshold for obesity were associated with self-identifying as obese or very overweight.

**Conclusions:**

The majority of the adult obese population of Great Britain do not identify themselves as either ‘obese’ or even ‘very overweight’. Public health initiatives to tackle obesity are likely to be hampered by this lack of recognition of weight status. It is important to understand whether moves to increase personal awareness of weight status in the obese population can facilitate beneficial behaviour change, and what role health professionals can play in increasing awareness of weight status in obese patients.

**Strengths and limitations of this study**

- We studied weight perceptions among obese adults in Great Britain in 2007 and 2012, using repeated, cross-sectional, population-based surveys. Data collection methods were the same at both time points. This approach provides access to a demographically and geographically diverse sample of obese adults.
- Weight self-perceptions are known to correspond poorly to clinical definitions. This study shows that the ‘normalisation’ of larger body sizes extends into the obese range and is increasing in women.
- Self-identification as ‘obese’ remained very low (<10%) between 2007 and 2012, and among obese women, self-identification as ‘very overweight’ sharply declined, despite extensive media and public health attention to the health risks of excess weight.
- Only survey participants whose self-reported heights and weights defined them as obese were included in these analyses. The self-report methodology is likely to mean that some obese participants were excluded due to underestimation of their body mass index.
- A higher proportion of women in the survey sample declined to provide height and weight information in 2012 than 2007, which may reflect increasing sensitivity surrounding issues of body weight.

## Introduction

Over two decades, studies from several different countries have demonstrated a decrease in the proportion of overweight adults who recognise that their weight places them in the overweight or obese categories.^1–3^ This could have serious consequences for those meeting the clinical definition of obesity; leaving them less likely to recognise the health implications of their body weight, or to make appropriate lifestyle changes or seek treatment.^4–6^

However, recent years have seen a dramatic increase in the profile of obesity as a public health problem. The search for effective ways to raise awareness of the problem of excess weight and encourage lifestyle change has resulted in a number of highly visible public health interventions in the UK.^7^ ^8^ Media coverage of the subject has similarly burgeoned.^9^ This increased attention to obesity and its associated health implications might be expected to have resulted in improved public knowledge and awareness; particularly among the obese population for whom the information should be most salient.

We therefore examined recognition of personal weight status in population-based samples of obese men and women from 2007 to 2012 in Great Britain. Since there is evidence that the term ‘obese’ can be perceived as derogatory, and many individuals whose body mass index (BMI) defines them as obese reject the term,^10^ ^11^ we also examined the extent to which obese adults self-identified with the less controversial term ‘very overweight’. In addition, we tested whether self-identification as very overweight or obese was associated with greater awareness of the BMI threshold for obesity.

## Methods

### Design and participants

Data for these analyses were taken from two commissioned commercial population surveys of British adults, carried out as part of the TNS/BMRB face-to-face omnibus surveys in May 2007 and March 2012. Data were collected using a two-stage random location sampling method. One hundred and forty-three sampling points were selected from across England, Wales and Scotland using a sampling frame stratified by government office region, social grade and rural/urban location. In each location, clusters of a minimum of 125 households, based on census enumeration districts, were randomly selected. Interviewers recruited participants in accordance with a quota system based on gender, children in the home and working status. Data weights were provided to match the sample to the British population. Surveys were conducted in the home, with one interviewee, aged above 16 years, randomly selected per household. The present analyses used data from respondents whose self-reported weight and height placed them in the obese range (BMI ≥30).

### Measures

#### Demographics

Demographic variables included in these analyses were age, sex and social grade. Social grade was classified according to the National Readership Survey occupational social grade classification system (2007) which has six categories. For multivariable analyses it was dichotomised into higher (ABC1: professional, managerial and supervisory) and lower social grade (C2DE: skilled and unskilled manual workers).

#### Anthropometric data

Weight and height were self-reported in metric or imperial units according to the respondent's preference. BMI was calculated using the standard formula (weight in kg/ height in m^2^).

#### Perceived weight

Respondents were asked to select a descriptor for their own body weight from the following list of options: *very underweight, underweight, about right, overweight, very overweight, obese*.

#### Knowledge of BMI

This was assessed with the question: ‘Have you ever heard of Body Mass Index’ (Yes/No), with a follow-up question to those who responded affirmatively: ‘Do you know what Body Mass Index is considered to be obese’. A response of 30 was classified as correct, and all other responses as incorrect.

### Data analysis

Analyses were carried out in SPSS/PASW V.18. t Tests and χ^2^ analyses were used to compare data from the 2007 and 2012 surveys. Data were weighted to be representative of adults aged 16+ in Great Britain, and weighted data were used for all analyses. Unique predictors of self-identification with either of the terms ‘very overweight’ or ‘obese’ were examined using logistic regression, with analyses carried out separately for men and women. Variables in the analysis were age, obesity grade, social grade, survey year and knowledge of BMI.

## Results

The full unweighted sample comprised 1998 respondents (895 men, 1103 women) in 2007, and 1986 (932 men, 1054 women) in 2012. In both surveys, the majority of respondents provided height and weight data allowing calculation of BMI: 1838 (92%) in 2007 and 1701 (86%) in 2012; although the proportion declining to give height or weight data was significantly higher in 2012 than 2007 (χ2=39.74 p<0.001). This was particularly marked among women. Analysis of cases with missing height and weight data showed that women declining to provide height or weight measurements in 2012 were somewhat younger (43.7 years vs 48.8 years t=−3.34 p<0.001), but did not differ significantly by social grade (p=0.260) or perceived weight (p=0.393). Of those providing height and weight data, 160 (18.8%) men and 182 (18.4%) women in 2007, and 166 (19.6%) men and 149 (17.4%) women in 2012 reported weights and heights corresponding to a BMI >30 kg/m^2^; defining the group of 657 obese respondents.

### Obese sample comparisons 2007–2012

The characteristics of the weighted male and female obese participants are shown in table 1. They were similar in anthropometric and demographic characteristics, with no significant differences in age, social grade or BMI across the two surveys in women. The male participant were slightly older in 2012 (49.7 years) than in 2007 (46.5 years; t=2.0 p=0.05), but did not differ by social grade or BMI.

**Table 1:** Demographic characteristics of obese men and women and their weight perceptions and BMI knowledge: 2007 and 2012

	Women						Men					
	2007		2012			p Value	2007		2012			p Value
	N=164		N=143				N=178		N=172			
Age (mean, SD)	50.2	(16.1)	52.7	(17.5)	t=−1.3	0.19	46.5	(14.9)	49.7	(15.2)	t=−2.0	<0.05
BMI (mean, SD)	35.1	(4.6)	34.3	(4.3)	t=1.72	0.09	33.5	(3.7)	34.3	(5.2)	t=−1.5	0.13
Social grade % (n)												
High (ABC1)	48.8	(80)	49.7	(71)	χ^2^=0.02	0.88	47.2	(84)	48.3	(83)	χ^2^=0.04	0.84
Low (C2DE)	51.2	(84)	50.3	(72)			52.8	(94)	51.7	(89)		
Describe your current weight % (n)												
Underweight/about right	1.8	(3)	5.6	(8)	χ^2^=10.61	<0.05	10.7	(19)	10.5	(18)	χ^2^=3.73	0.29
Overweight	48.2	(79)	60.8	(87)			62.4	(111)	66.3	(114)		
Very overweight	37.2	(61)	23.1	(33)			23.0	(41)	16.3	(28)		
Obese	12.8	(21)	10.5	(15)			3.9	(7)	7.0	(12)		
BMI knowledge % (n)												
Heard of BMI	75.6	(124)	79.7	(114)	χ^2^=0.74	0.39	73.6	(131)	76.2	(131)	χ^2^=0.31	0.58
Correctly identify BMI ‘obese’	12.2	(20)	8.4	(12)	χ^2^=1.18	0.28	5.1	(9)	7.0	(12)	χ^2^=0.57	0.45

Weighted base: women=307 men=350.

BMI, body mass index.

### Changes in weight perceptions

**Figure 1:**
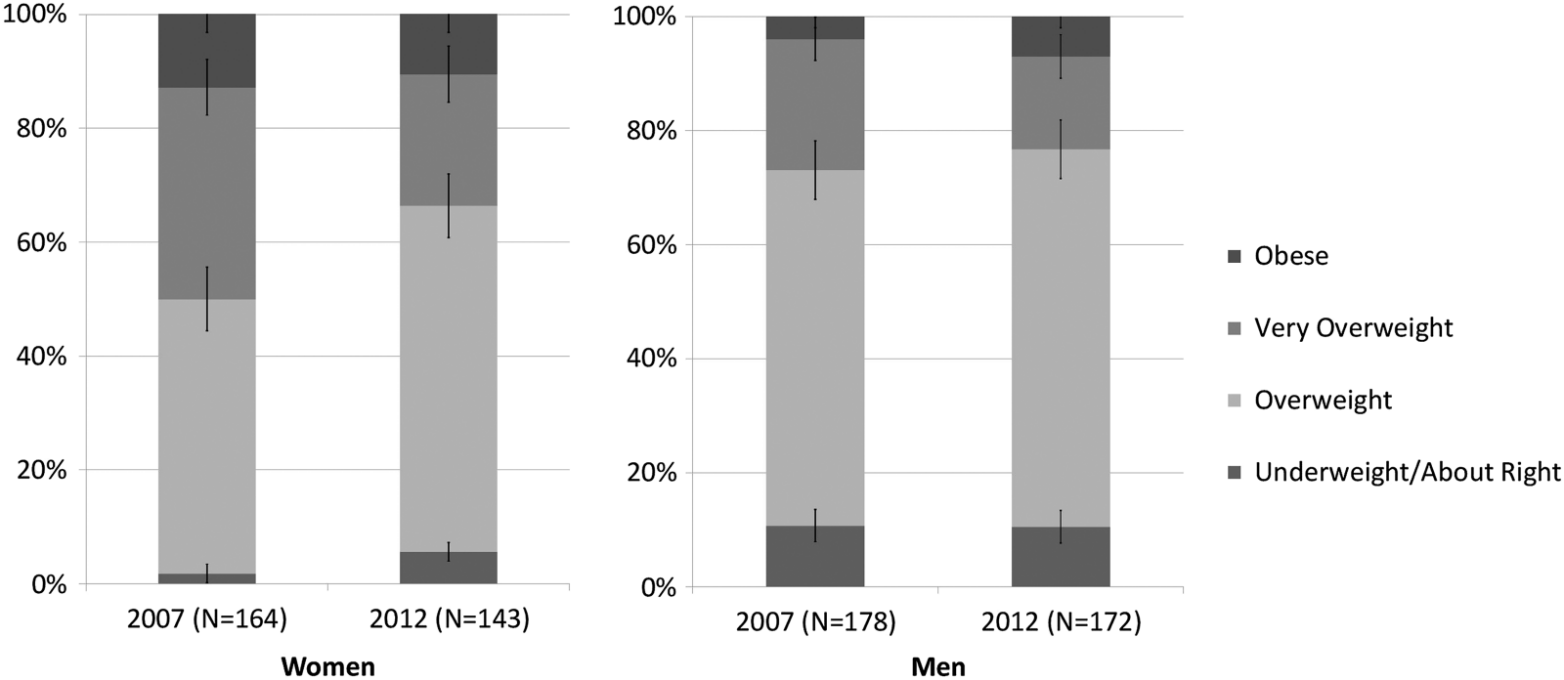
Perceived weight in obese adults in Britain.

In women, weight perceptions changed significantly between 2007 and 2012 (χ^2^=10.6 p<0.05; table 1), reflecting a substantial decline in self-identification with the terms ‘obese’ or ‘very overweight’ in favour of either ‘overweight’ or ‘about right’ (figure 1). Endorsement of the term ‘obese’ was low at both time points (12.8% in 2007 and 10.5% in 2012) and did not change significantly (p=0.53). Owing to the small numbers endorsing this clinically accurate descriptor for their weight, those perceiving themselves to be ‘very overweight’ were combined with the perceived ‘obese’ group for subsequent analyses. In 2007, 50% of obese women endorsed either ‘very overweight’ or ‘obese’, compared with just 33.6% in 2012, indicating a significant decrease in recognition of substantial excess body weight (χ^2^=8.45 p<0.01).

Among men, differences in weight perceptions between the two surveys did not reach statistical significance (χ^2^=3.73 p=0.29). Very few men endorsed the term ‘obese’ at either time point (3.9% in 2007 and 7% in 2012). When those endorsing ‘very overweight’ were combined with those endorsing ‘obese’, recognition of substantial excess weight was 26.9% in 2007 and 23.3 in 2012.

### BMI knowledge

Around three quarters of participants said they had heard of BMI at each time point (table 1), with no significant change among either women (75.6% in 2007 and 79.7% in 2012; χ^2^=0.74, p=.39) or men (73.6% in 2007 and 76.2% in 2012; χ^2^=0.31, p=0.58). However, the majority did not know the correct BMI threshold for obesity. Among women, 12.2% identified the BMI threshold for obesity in 2007 and 8.4% in 2012. Among men, the corresponding figures were 5.1% in 2007 and 7% in 2012. There were no significant changes between the two time points for either women (χ^2^=1.18 p=0.28) or men (χ^2^=0.57, p=0.45).

### Predictors of accurate weight perception

Factors associated with recognition of substantial excess weight were examined using multiple logistic regression in the combined 2007 and 2012 data sets with survey year as an independent variable. We defined recognition of substantial excess weight as self-identification as either ‘very overweight’ or ‘obese’ (table 2).

**Table 2:** Predictors of self-perceived weight (very overweight/obese) among obese British adults (multivariable analysis)

	Women			Men		
	%*	OR (95% CI)	p Value	%*	OR (95% CI)	p Value
Age		0.99 (0.98 to 1.01)	0.28		1.00 (0.98 to 1.02)	0.81
Obesity grade						
Grade 1 (BMI 30–<35)	33.2	1	<0.001	19.6	1	<0.001
Grades 2/3 (BMI ≥35)	58.6	3.27 (1.95 to 5.48)		42.4	3.26 (1.86 to 5.72)	
Survey year						
2007	50.0	1	<0.05	27.0	1	0.14
2012	33.6	0.53 (0.32 to 0.88)		23.3	0.68 (0.40 to 1.14)	
Social grade						
Higher (ABC1)	47.3	1	0.09	24.6	1	0.49
Lower (C2DE)	37.8	0.65 (0.39 to 1.07)		25.7	1.21 (0.71 to 2.04)	
BMI knowledge†						
Incorrect/not known	40.0	1	<0.05	23.9	1	<0.05
Correct	62.5	2.68 (1.16 to 5.19)		45.0	3.19 (1.18 to 8.61)	

*Indicates percentage within each group perceiving themselves to be very overweight/obese.

†BMI knowledge: ‘Do you know what Body Mass Index is considered to be obese?’ (BMI=30 vs all other responses).

Weighted data. Base: women=307, men=350, All=657.

BMI, body mass index.

Among women, self-identification as ‘very overweight’ or ‘obese’ was independently associated with higher BMI (OR=3.27 p<0.001), such that 58.6% of those with grade 2/3 obesity (BMI >35) identified as ‘very overweight’ or ‘obese’ vs 33.2% of those with grade one obesity. Women who knew the BMI threshold for obesity were also more likely to identify as ‘very overweight’ or ‘obese’ (62.5%) than those who did not (40.0%; OR=2.68 p<0.05). Women were less likely to identify themselves as ‘very overweight’ or ‘obese’ in 2012 than in 2007 (33.6% vs 50.0%; OR=0.53 p<0.05). There were no significant independent associations with age (p=0.28) or social grade (p=0.09) in women.

Men were more likely to describe themselves as ‘very overweight’ or ‘obese’ if they had a higher BMI, such that 42.4% of men with grade 2/3 obesity self-identified as ‘very overweight’ or ‘obese’ compared with 19.6% of men with grade one obesity (OR=3.26 p<0.001). They were also more likely to describe themselves as ‘very overweight’ or ‘obese’ if they knew the BMI threshold for obesity (45%) than if they did not (23.9%; OR=3.19 p<0.05). There were no significant independent associations with age (p=0.81), social grade (p=0.49) or survey year (p=0.14) in men.

## Discussion

We used data from two population-based surveys with the same data collection methods carried out in 2007 and 2012, to examine weight perceptions in the obese population of Great Britain. We hypothesised that the increasing media and public health focus on obesity over this time would have resulted in greater awareness of excess weight status by obese adults. Survey respondents were selected for analysis on the basis of providing self-reported weight and height data that identified them as clinically obese (BMI >30) and were asked to choose a term to describe their body weight. The response options for self-perceived excess body weight included both ‘obese’ and ‘very overweight’. Few previous studies examining public weight perceptions have included the term ‘obese’; with most offering only ‘overweight’ or ‘very overweight’ as response options.^12–17^ The present results therefore provide a level of benchmarking.

The results showed very low levels of self-identification with the term ‘obese’ at either time point, and among either men or women, and no significant changes in identification with this term over time. Clearly there is substantial continuing resistance among the obese population in Britain to identifying themselves as obese.

Several previous studies have shown that the term ‘obese’ is widely perceived as stigmatising, and might be rejected as a self-descriptor for that reason.^10^ ^11^ We therefore also examined trends in acceptance of the less controversial descriptor ‘very overweight’. Acceptance of this term would suggest an appreciation that a healthy weight is exceeded by some margin. However, among women there was a substantial *decrease* in endorsement of the term ‘very overweight’ from 2007 to 2012, and corresponding increases in the proportion of obese women describing themselves as either ‘overweight’ or ‘about right’. This suggests weight misperception rather than simply a rejection of the term ‘obese’. Among men self-perception as either obese or very overweight was slightly lower in 2012 than 2007, but the difference was not statistically significant.

There was also no improvement between 2007 and 2012 in knowledge of the BMI threshold for obesity, which remained very low at around 10% in women and less in men. Knowledge of the BMI threshold for obesity was a significant predictor of more accurate weight perceptions in men and women in multivariate analyses. This may imply that improving knowledge could increase accuracy of weight perception, although better knowledge could be a marker for greater engagement with weight and health issues.

This study has limitations. Although the data were taken from population-based surveys, the sample was not stratified for body weight, and so the obese subsample may not represent the UK obese population. The same methodology was used at both time points, but a higher proportion of interviewees declined to give height and weight information in 2012 than in 2007, which may reflect increasing sensitivity surrounding issues of body weight. This was particularly marked among younger women. Nonetheless, the sample was drawn from all socioeconomic groups, ages, and geographical areas, and as such, is likely to give a valid indication of trends in weight perceptions.

The use of self-reported anthropometric data means that true height was likely to be overestimated and true weight underestimated.^18^ ^19^ Both average BMI and the proportion of the population who are overweight or obese will therefore be underestimated; resulting in exclusion of some obese people. Finally, the very small number of participants endorsing the term ‘obese’ limits the interpretation of changes in acceptance of this term.

The trend towards ‘normalisation’ of a body size in the ‘obese’ range appears to be continuing, at least among women. Social comparison processes are likely to play a part,^20^ although increases in population weights cannot altogether explain this continuing trend, as the prevalence of adult obesity has changed little over this time period.^21–23^ However, longer exposure to the new weight profile of the population may increase familiarity with larger body sizes, and normalisation of larger body weights may therefore still be in progress.

The framing of obesity-related news stories can also contribute to normalisation of obesity. Analyses of media coverage of obesity-related stories have highlighted the extreme, stereotyped, and stigmatising images of obesity used to illustrate such stories, showing that they often feature cases of morbid obesity, which do not represent the appearance of the majority of obese individuals.^24–26^ This could contribute to lower recognition of obesity among those whose weight is at the lower end of the obesity spectrum, as seen in this study.

This study highlights a continuing disconnection between the obese population and the medical community regarding definitions of obesity. Health professionals may have a role to play in increasing awareness,^27^ although concern that use of the term ‘obese’ is stigmatising, and the difficulty of broaching this sensitive subject in consultation with patients who are not seeking help with their weight, have been identified as barriers.^28^ Effective channels of communication are needed in order to counteract a perception among obese individuals that obesity is an extreme state and the term ‘obese’ does not apply to them. Otherwise, increasing numbers of those whose weight represents a risk to their health are likely to remain unaware of the personal relevance of weight-related health messages.
